# Biomechanical comparison of a new stand-alone anterior lumbar interbody fusion cage with established fixation techniques – a three-dimensional finite element analysis

**DOI:** 10.1186/1471-2474-9-88

**Published:** 2008-06-18

**Authors:** Shih-Hao Chen, Ching-Lung Tai, Chien-Yu Lin, Pang-Hsing Hsieh, Weng-Pin Chen

**Affiliations:** 1 Department of Orthopaedics, Tzu-Chi General Hospital, Taichung, Taiwan; 2 Graduate Institute of Medical Mechatronics, Department of Mechanical Engineering, Chang Gung University, Taoyuan, Taiwan; 3 Department of Biomedical Engineering, Chung Yuan Christian University, Chungli, Taiwan; 4 Department of Orthopaedic Surgery, Chang Gung Memorial Hospital, Taoyuan, Taiwan

## Abstract

**Background:**

Initial promise of a stand-alone interbody fusion cage to treat chronic back pain and restore disc height has not been realized. In some instances, a posterior spinal fixation has been used to enhance stability and increase fusion rate. In this manuscript, a new stand-alone cage is compared with conventional fixation methods based on the finite element analysis, with a focus on investigating cage-bone interface mechanics and stress distribution on the adjacent tissues.

**Methods:**

Three trapezoid 8° interbody fusion cage models (dual paralleled cages, a single large cage, or a two-part cage consisting of a trapezoid box and threaded cylinder) were created with or without pedicle screws fixation to investigate the relative importance of the screws on the spinal segmental response. The contact stress on the facet joint, slip displacement of the cage on the endplate, and rotational angle of the upper vertebra were measured under different loading conditions.

**Results:**

Simulation results demonstrated less facet stress and slip displacement with the maximal contact on the cage-bone interface. A stand-alone two-part cage had good slip behavior under compression, flexion, extension, lateral bending and torsion, as compared with the other two interbody cages, even with the additional posterior fixation. However, the two-part cage had the lowest rotational angles under flexion and torsion, but had no differences under extension and lateral bending.

**Conclusion:**

The biomechanical benefit of a stand-alone two-part fusion cage can be justified. This device provided the stability required for interbody fusion, which supports clinical trials of the cage as an alternative to circumferential fixations.

## Background

Lumbar interbody cages are an improvement in spinal fusion that facilitate stabilization of the motion of segments and relieve discogenic back pain. They favor load transmission via the anterior column, annular fiber tensioning, restoration of the disc height and lordosis and have the least demands on bone graft volume [1-4]. The success of a fusion cage insertion, in addition to the biological factors, may depend upon other mechanical parameters, including the material properties of the vertebrae, the geometry of the implants, and the interface between the cage and the bone [5-7]. Although initial stability of the interbody spacers insertion is a requirement for successful fusion, the load transmission and its effect on the tissues adjacent to the fusion cage also play an essential role, which is not easily detectable with experimental tests [2,7,8]. Implantation of a single anterior interbody cage in a functional spinal unit has been investigated using finite element analysis (FEA) to reveal the altered load transfer and the neighboring structural change in relation to the peak stress distribution on the cage-bone contact interface [9,10]. Further examination of the stabilization effects of several fusion cages on the same specimen under different loading conditions will provide a better insight into the amount to which certain factors may influence the clinical outcomes.

Conventional cage designs have either cylindrical or rectangular shapes, thick walls, and a hollow interior space that contains grafting materials. Cylindrical cages have threads along their entire length, whereas rectangular cages have serrated anchors on the upper and lower surfaces. The rigid hollow design of fusion cages guarantees sufficient construct stiffness in arthrodesis and affords a substantial stability for the motion segments after spinal surgery, as well as shielding stress on the implanted graft [11,12]. The stability of a cage-buttressed fusion relies on the strong apophyseal part of the endplate for support, as well as the neighboring vertebrae ensuring sufficient density in the peripheral region to tolerate the alternation of load transfer following cage insertion [7,13]. Failure of the implant-endplate interface may occur in an osteoporotic spine with cage subsidence, migration and subsequent loss of disc height [6,7,10]. The anterior stand-alone traditional cage has been reported to reduce intervertebral motion in flexion and lateral bending, while no stabilization was achieved during extension and axial rotation [2,5]. Although supplementation of posterior fixation diminishes residual segmental mobility and preserves lumbar lordosis, the optimal construct performance and all cage-bone interface mechanics have yet to be determined. A newly designed two-part fusion cage consisting of a rectangular frame that accommodates a threaded cylinder holding bone graft material was developed. A biomechanical comparison between the two-part cage and the conventional interbody spacers will be completed. The purpose of this study was to use FEA models to investigate the cage-bone interface mechanics and stress distribution on the adjacent tissues after insertion of several interbody fusion cages with or without the supplementary posterior fixation. Based on the parametric measurement of contact stress on the facet joint, maximum slip displacement of the implants on the endplate, and rotational angle of the upper vertebra in relation to the peak stress of contact site, the biomechanical differences of several implanted constructs were assessed under different loading conditions.

## Methods

### Generation of L4-5 intact finite element model

A 27 year-old male with paraplegia scheduled for a computed tomography (CT) examination of the lumbar spine was recruited. A one-millimeter scan interval was used from the L4 to L5 vertebrae in the transverse direction and the data files were transferred to a personal computer for image processing. The contours of the cortical and cancellous bone were used to generate the solid model in the Solid Works CAD software (Solid Works Corp., Boston, U.S.A.). The surface models of the L4-5 vertebrae were transferred to a finite element pre-processing program – Mentat (MSC Software Corp., Los Angeles, U.S.A.) and the finite element mesh of the intact L4-5 vertebrae was generated with 10-node tetrahedral elements. The determination of the facet joints was difficult since there were only a few CT images across the facet joint. Therefore, the orientations and gaps of the facet joints were manually created according to literature [14]. The facet joints were assumed to be frictionless, had a gap of 0.5 mm, and could only transmit compressive force [15].

The dimension and position of the intervertebral disc were determined from the adjacent endplates. The nucleus pulposus and annulus fibrosis were modeled with solid elements with linear elastic material properties. All seven ligaments (anterior and posterior longitudinal ligaments, ligamentum flavum, facet capsular ligament, interspinous ligament, supraspinous ligament and intertransverse ligament) were included in the finite element model (FEM). These elements were modeled as tension-only cable elements with linear elastic behavior. The insertion points, cross-sectional areas, and material properties were adopted from anatomy textbook and various reports. The material properties for the different parts of the model were assigned according to previous literature as shown in Table 1[16-18]

**Table 1:** Material properties specified in the finite element models

Material	Elastic modulus (MPa)	Poisson ratio	Total element number	Cross section area (mm^2^)
Cancellous bone	100	0.2	18185	-
Cortical bone	12000	0.3	6512	-
Endplate	1000	0.4	2361	-
Nucleus pulposus	10	0.4	1281	
Posterior elements	3500	0.25	16587	-
Pedicle screw/cage	110000	0.3	6388	-
***Annulus fibre layers***				
Outermost	550	0.3	-	0.7
Second	495	0.3	-	0.63
Third	440	0.3	-	0.55
Fourth	420	0.3	-	0.49
Fifth	385	0.3	-	0.41
Innermost	360	0.3	-	0.30
***Ligaments***				
Lig. long. posterius	70	-	-	20
Lig. flava	50	-	-	60
Lig. intertransversia	50	-	-	10
Lig. interspinalia	28	-	-	35.5
Lig. supraspinalia	28	-	-	35.5
Lig. capsulae	20	-	-	40

To investigate the interface mechanics between the implant and bone, the endplate density, pedicle diameter, facet gap distance, and elastic modulus of annulus fibers and ligaments were considered to be identical. The intact finite element model of the L4-5 functional spinal unit consisted of 51322 elements.

### Generation of models implanted with interbody spacers and posterior instrumentation

**Figure 1:**
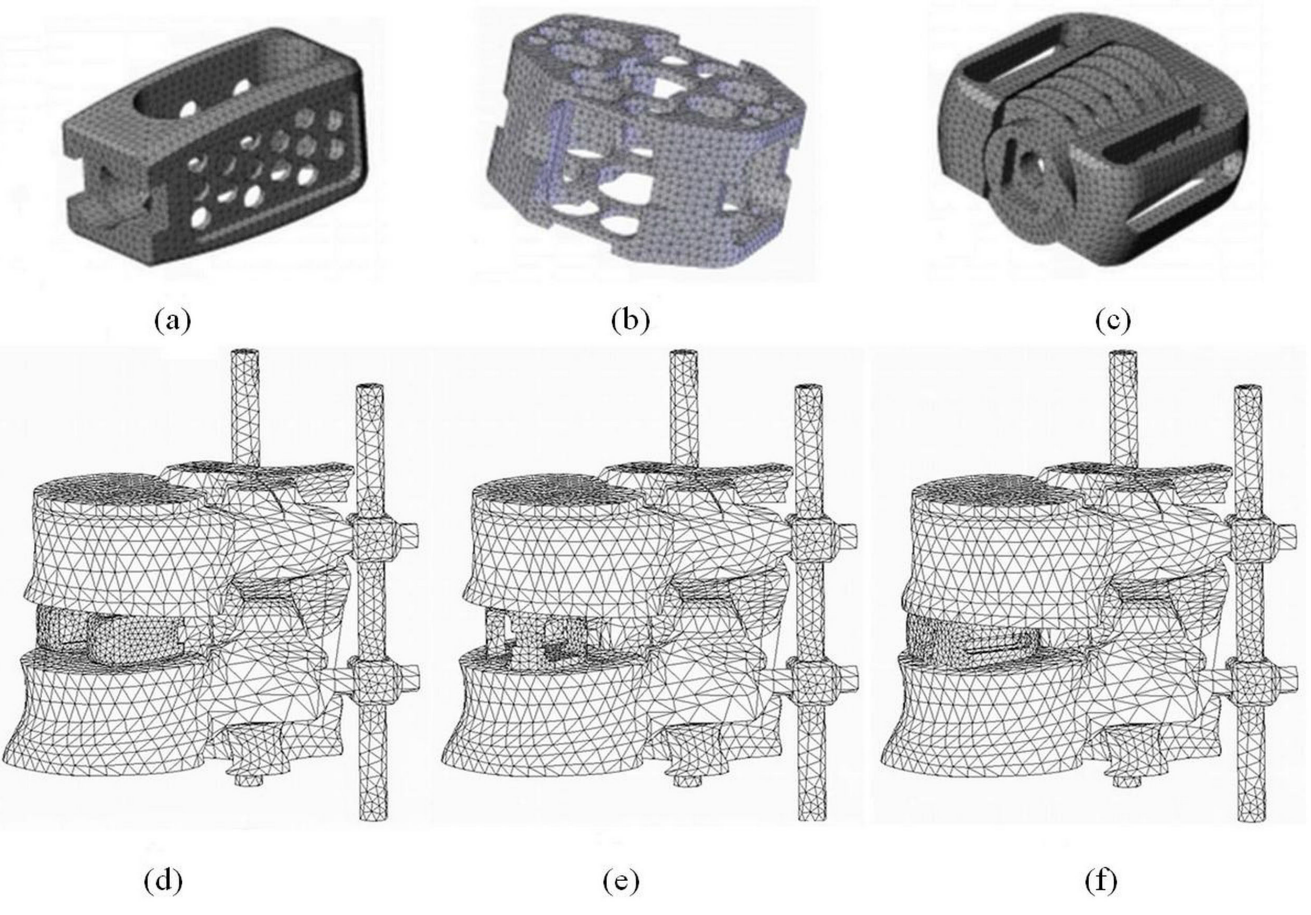
**Finite element models of three interbody cages:** (a) dual paralleled cage; (b) single large cage; (c) two-part cage, and (d, e, f) each inserted in the L4–L5 motion segment supplemented with posterior instrumentations.

Using the anterior spinal approach and removal of the anterior longitudinal ligament, the following three different spacers were inserted to stabilize the anterior column for interbody fusion: (A) the Contact Fusion Cage (Stratec, Oberdorf, Switzerland) is a small rectangular cage in dual-parallel position, denoted as "DPC"; (B) the SynCage (Mathys Medical Ltd., Bettlach, Switzerland) is a single monobloc, box-shaped large cage, denoted as "SLC"; (C) the Stabilis (Stryker, Michigan, U.S.A.) device is made of two distinct parts – an anatomically rectangular frame with a threaded, cylindrical delivery unit of bone graft and is denoted as "TPC". The three cages are made of titanium alloy and have known material properties. The appropriate size of trapezoid 8° cage was chosen according to the space between the vertebrae, as proposed by the manufacturer to restore lumbar lordosis and disc height. Accordingly, a modulus of 110 GPa and Poisson's ratio of 0.3 were defined for titanium alloy (Table 1) [15]. The FEMs of the three different cages are shown in Figure 1a–c, respectively. The FEMs of the L4-5 motion segment implanted with three different cages are shown in Figure 1d–f, respectively.

The residual annulus of the operated intervertebral disc was ignored because it was likely to be compromised significantly by the anterior surgical procedure and because the stiffness of the remaining tissue would be very low as compared to that of the interbody cage. Bone graft and bone ingrowth into the hollow interior space were not modeled in this study.

### Contact definition

The interfaces between the cage and endplate were modeled with contact bodies. Slip displacement was defined as the relative micromotion on the implant-bone interface by calculating the length between two adjacent contact nodes on the contact bodies. For the definition of interaction between the implant and the bone, a Coulomb friction contact algorithm was used to model the force transmission between implant and vertebral endplate. The coefficient of friction for the interaction was set to 0.4 for the benchmark case [19]. Commercially available implants often have serrated contact surfaces, creating higher effective friction coefficients than would be expected with smooth contact surfaces, which were modeled in this study. The facet joints were treated as nonlinear, 3-dimensional contact interfaces, including friction on the joint surfaces. Finite-sliding interaction was defined and allowed any arbitrary motion of the surfaces, i.e. separation, sliding and rotation.

### Loading and boundary conditions

The loading conditions were applied on the superior surface of the L4 with 150 N of compressive pre-load, together with four different kinds of 10 N-m moments to simulate the following motions: (1) flexion, (2) extension, (3) right lateral bending, and (4) torsion [20]. To homogenize the load influence, the forces were distributed to the nodes on the superior surface of L4. The inferior surface of L5 was constrained in all direction. The selection of these loads simulated situations in an in vitro experimental study, allowing for validation FEA output of an intact spine model [8,10,21,22]. The commercially available FEA software MARC (MSC Software Corp., Los Angels, USA) was used for these analyses. Analyses were performed using the computing facilities at the National Center for High-Performance Computing (NCHC, Hsin-Chu, Taiwan) via internet connection. Analysis results were retrieved back and processed on a local personal computer. All analysis results for the models with interbody spacers were compared with those of the intact one.

## Results

### Convergences of FEM

The convergences of the FEMs were justified by the total strain energy of the structures. Four models with different numbers of elements and nodes were created to perform the convergence test, and the results of the total strain energy for the four models were all within 5% difference. In this study, the model with the finest mesh was used and the convergence of the FEMs was demonstrated from the above procedures.

### Validation of the intact FEM

**Figure 2:**
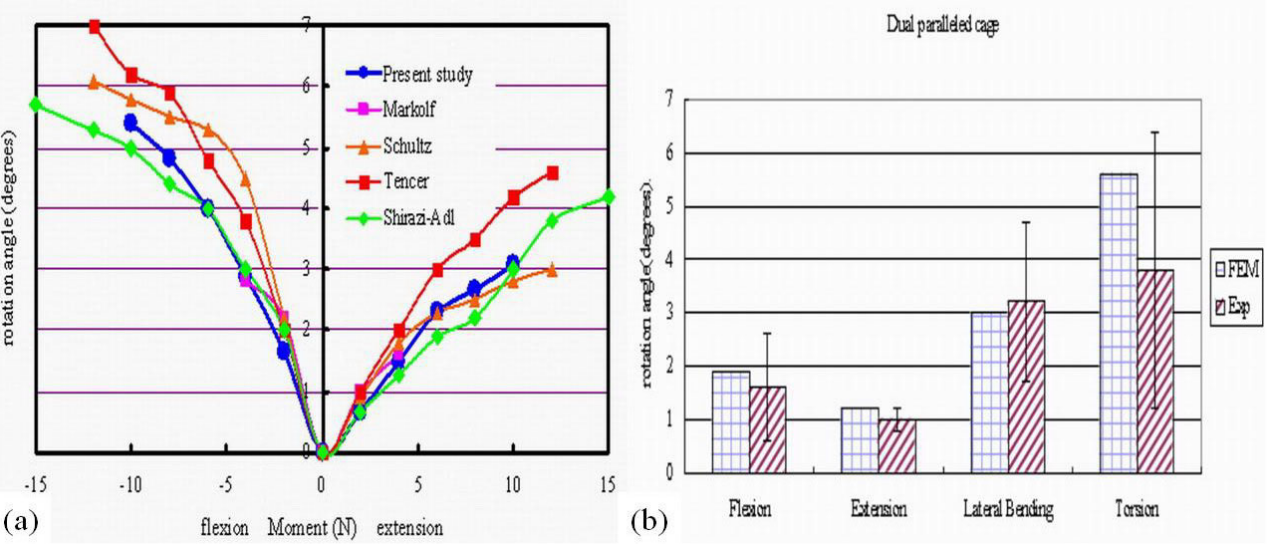
**(a)** Validation of our intact finite element model, as compared with Markolf, Schultz, Tencer, Shirazi-Adl et al studies; **(b)** Validation of our finite element model with dual paralleled cage insertion, as compared with Tsantrizos et al. experimental study.

We validated the intact FEM by comparing the flexion and extension angles with those of previously published experimental studies [23,24]. The previous studies had indicated that the flexion and the extension angles ranged between 5° – 6.2° and 2.8° – 4.2°, respectively, under a 10 N-m flexion or extension moment. In current study, the flexion angle was 5.4° under a 10 N-m flexion moment, and the extension angle was 3.1° under a 10 N-m extension moment. These data were closely correlated with the results of past studies (Figure 2a).

### Validation of the FEM with cage insertion

Initially, our FEM with dual-paralleled cage insertion was validated by comparing the rotational angle of vertebrae with those reported using Tsantrizos, experimental setup, which applied the following moments: (1) 4.0 N-m flexion-extension moment; (2) 4.0 N-m axial rotation moment; (3) 8.0 N-m lateral bending moment. All loading conditions were applied with **a **200 N preload (Figure 2b) [25]. In addition, we found that the relative micromotion at the implant-bone interface increased with the increasing compression force (150N–600N), and with the decreasing friction coefficient (0.4–0.2) [19]. All the calculated data were consistent with the in vitro experimental reports.

### Stresses of interbody cages on vertebral endplates

**Figure 3:**
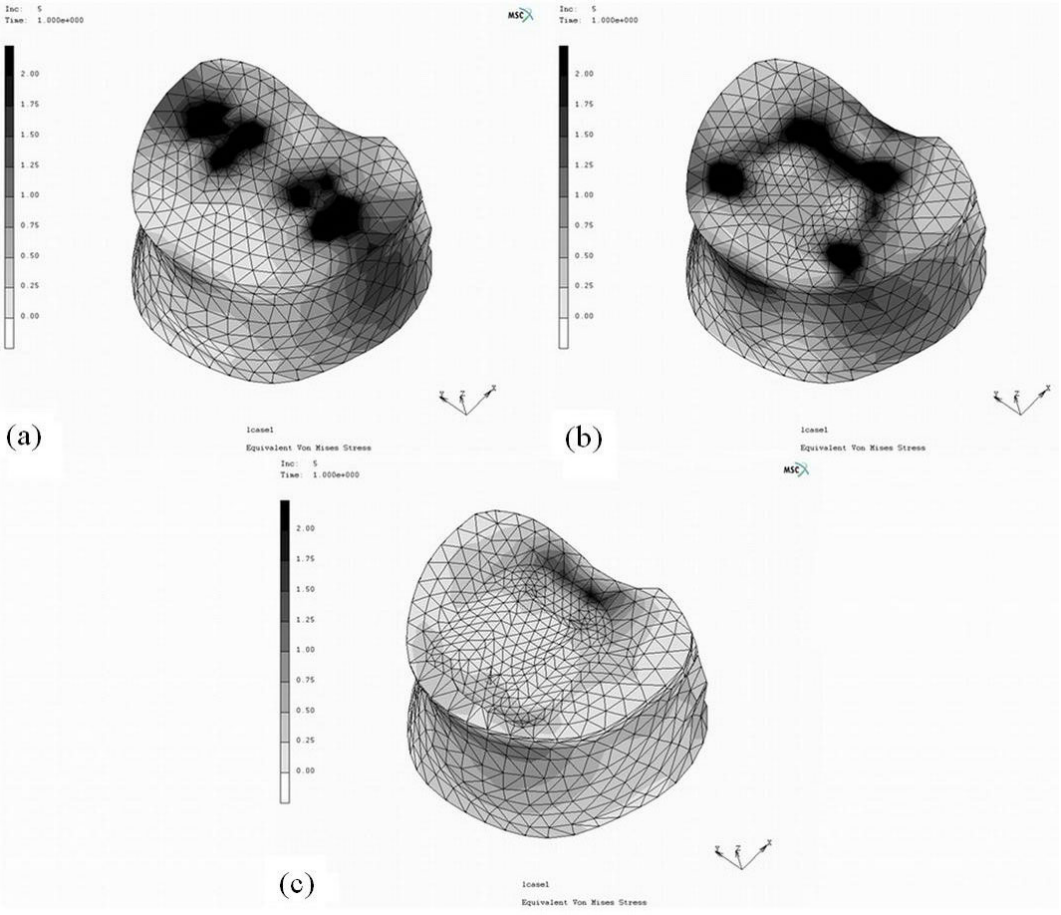
**The maximum von Mises stress distribution on the L5 upper endplate after insertion of various interbody cages:** (a) dual paralleled cage; (b) single large cage; (c) two-part cage.

To simplify the analytic procedures, identical cancellous bone density of 100 MPa was defined in our study to evaluate the peak stress of the selected cages designs on the vertebral endplate. With the consistent lordotic angle and maximal contact of the cage-bone interface, the peak stress of the DPC was mapped on the whole contact area of the endplate, the SLC on the periphery of posterior interface, and the TPC on the central edge of posterior interface (Figure 3). The cage insertions, with different designs, illustrated significantly different peak stress distribution on the cage-bone interface. Examples for the percentile differences of the maximum von Mises stress on the L5 superior endplate were calculated as (σ_DPC_-σ_TPC_)/σ_DPC _or (σ_SLC_-σ_TPC_)σ_SLC_. The maximum stress of the endplate after the TPC insertion decreased by 60% and 23% as compared with that after the DPC and SLC insertion, respectively.

### Facet contact stresses after interbody cages insertion

**Figure 4:**
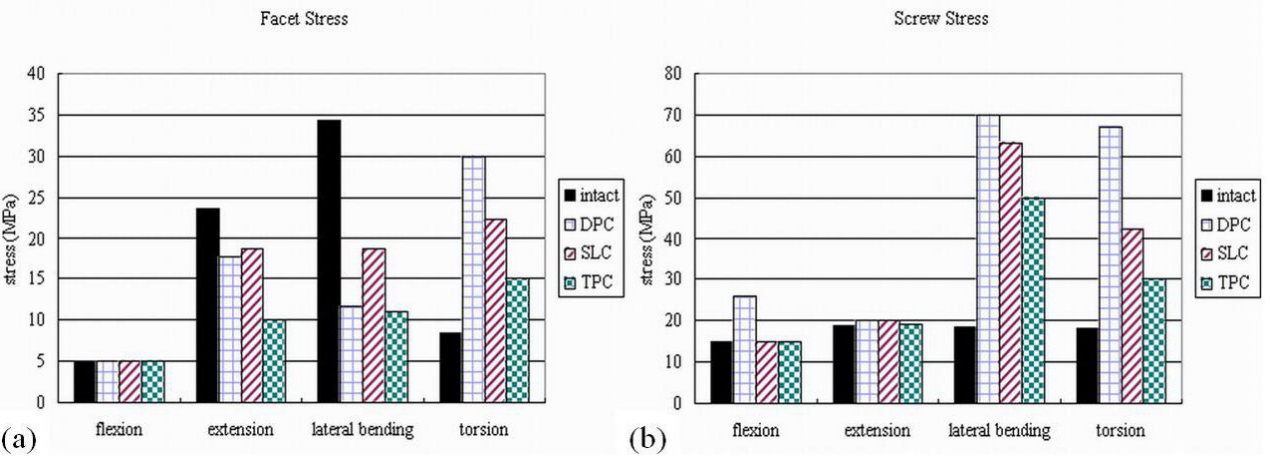
**(a)** Facet contact stresses values before and after interbody cages insertion; **(b)** Supplemented screw stresses values, separately, before and after interbody cages insertion.

Under flexion-extension, lateral bending and torsion (10 N-m moment loading) conditions, the facet contact stress values in the FEM with cage insertion were calculated (Figure 4a). The predicted facet stress values under extension, lateral bending and torsion loadings were larger than those under flexion in all cage groups. Besides, the predicted facet stress values after DPC and SLC insertion were larger than those after TPC insertion. When supplemented with posterior pedicle screw fixation, most of the facet stress was shared by the supplemented implants. The stress values on the screws were comparable among the intact, DPC, SLC and TPC groups under flexion and extension loadings (Figure 4b). On the other hand, under lateral bending and torsion, the stress values on the screw of DPC, SLC and TPC groups were larger than the intact group.

### Slip distance of the implant-bone interface after interbody cage insertion

**Figure 5:**
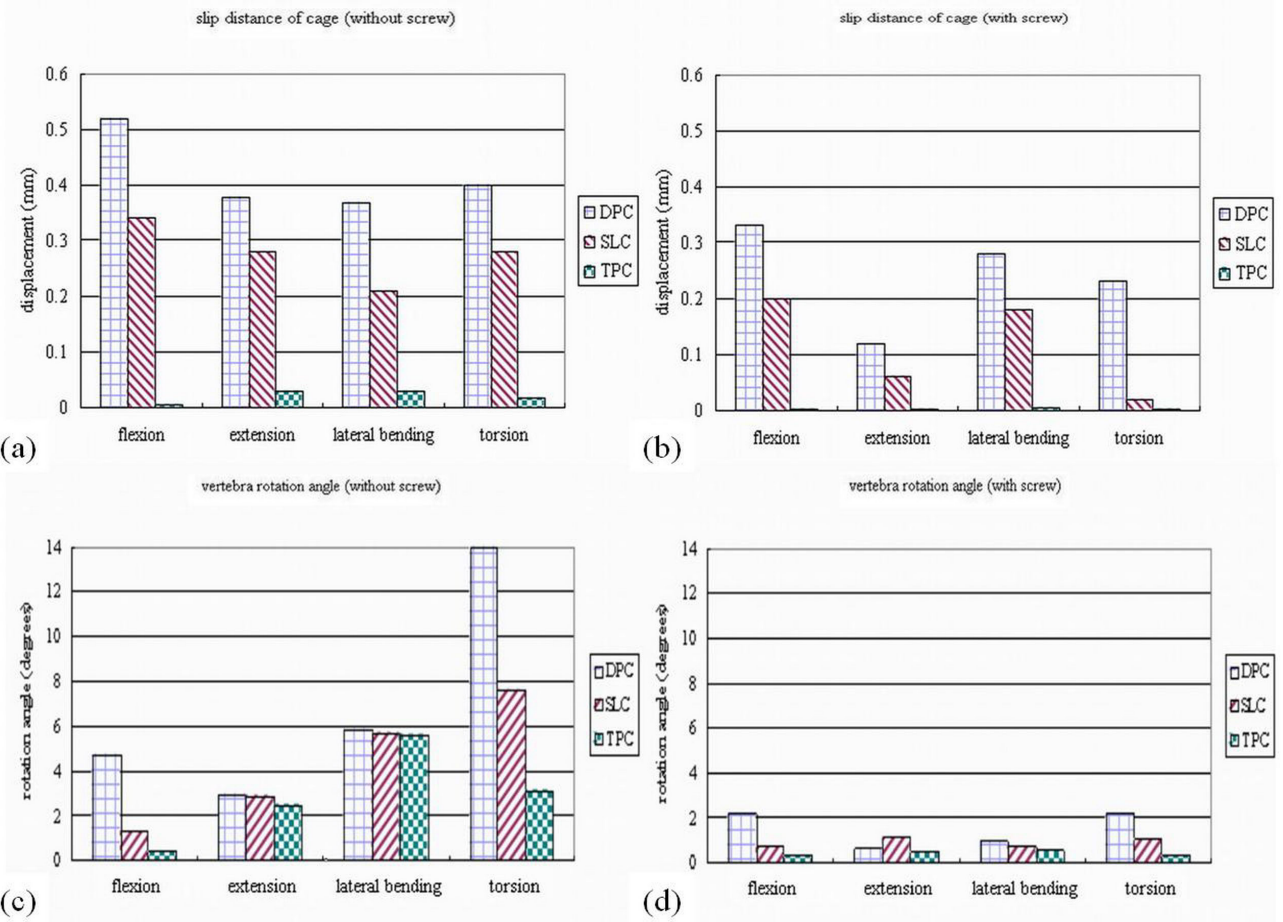
**Slip distance of the implant-bone interface after interbody cages insertion:** (a) without pedicle screw supplementation; (b) with pedicle screw supplementation. Rotational angle of the upper vertebra after interbody cage insertion; (c) without pedicle screw supplementation; (d) with pedicle screw supplementation.

Relative micromotion on the implant-bone interface was noted at the peripheral edges under axial compression, which increased with the addition of flexion, extension, lateral bending and torsion moments to the compressive preload [19]. In our study, with the setting of determined cancellous bone density, loading force, and friction coefficient of 0.4, the slip displacement values on the cage-bone interface were calculated in various loading conditions. Under each loading condition, the predicted displacement values after TPC insertion were smaller than those after DPC and SLC insertion (Figure 5a). When supplemented with posterior fixation, the displacement values decreased by 25–40% under flexion, lateral bending and torsion loadings and decreased by 70% under extension among the DPC and SLC groups. In TPC group with posterior fixation, the predicted displacement value decreased to zero level under all loading conditions (Figure 5b). Most likely, there was no significant effect of posterior fixation upon the slip behavior of TPC insertion group.

### Rotational angle of the upper vertebra after interbody cage insertion

With the consistent segmental lordosis and maximal contact of the cage-bone interface, the rotational angle of the upper vertebra was calculated, as compared to the intact model, under various loading conditions. Under flexion and torsion loading conditions, the predicted rotational angles after the TPC insertion were smaller than those after the DPC and SLC insertion (Figure 5c). However, there was no difference in rotational angle under lateral bending and extension, which might be attributed to the threaded cylinder incorporated in the TPC group. The threaded cylinder had no prominent effect on lateral bending and extension moments. When supplemented with posterior fixation, the rotational angle decreased by 80–90% under lateral bending and decreased by 60–80% under extension among the DPC, SLC and TPC groups (Figure 5d). Results were similar in all cage groups with or without supplementary posterior fixation under lateral bending and extension in spite of cage designs.

## Discussion

The introduction of interbody cages for spinal fusion has been a promising innovation; nevertheless, there is ongoing debate regarding the necessary conditions for successful fusion [1,4]. The influence of implant designs, surgical approach, additional posterior instrumentation and bone mineral density on stiffness, compressive strength and three-dimensional flexibility of the spinal units under static and cyclic loading have been investigated [2,5,7,11,13]. This study used FEA to investigate the interface mechanics and deformation levels in a range of various loading conditions among three trapezoid cage systems. In our FEMs with cage insertion, similar to that of Kim's report [19], micromotion at the cage-bone interface increased with applied load and was sensitive to the friction coefficient. Additionally, the parameters of facet contact stress, cage stress on the endplate and rotational angle of the upper vertebra were evaluated under different loading conditions in this study. In the event of physiological loading and maximal contact on the cage-bone interface, a stand-alone two-part fusion cage model has minimal slip displacement and rotational angle under compression, flexion, extension, lateral bending and torsion, as compared with the traditional cages, even with the additional posterior fixation. Conceptually, the adjacent vertebrae with sufficient density in the peripheral regions tolerate the above alteration following cage insertion, and an adequate remodeling for fusion is subsequently achieved.

Relative micromotion on the cage-bone interface can hinder bone growth into the surface pores of an interbody cage and eventually induce endplate failures with cage subsidence [9,10,12,18]. Interface mechanics provides insight and interpolation for the observed performance of established fixations and a point of departure for design improvements. A FEA study is an appropriate tool for such a purpose, allowing us to repeat experiments, change parameters, and analyze the effect of a single component within the designed constructs [9,10,19]. In the current FEMs with cage insertion under coupled loadings, complete contact on the cage-bone interfaces was assumed; therefore, the computed slip behaviors would be underestimated to avoid local bone failure from stress concentration. In addition, the slip distance of the cage-bone interface and the rotational angle of the upper vertebra were non-uniform, because of compressive indentation induced by body weight. The quantified amount of slip distance and rotational angle in the current two-part cage models is a critical advantage for the varying characteristics of traditional interbody spacers. Biomechanical benefit of the stand-alone two-part cage is justifiable in this study. Under flexion or torsion with a preload on the lordotic lumbar spine, the slip distance of the cages and rotational angle of the vertebrae measured at the anterior contact edges were smallest in the two-part cage group. However, under lateral bending or extension with a preload, there was no difference in the rotational angle among all cage models. Theoretically, the threaded cylinder incorporated in the trapezoid box had no prominent effect on the lateral bending and extension moments. A design modification of stand-alone cages using the divergent orientation of vertebral fixation incorporated in the box had been considered to overcome lateral bending and extension loadings [26]. However, as compressive load increased, improvement in the cage-bone contact and bone density of the adjacent vertebrae would overcome excessive cage micromotion on the endplate and lead to cage stabilization. Furthermore, age-related changes in the mechanical properties of the annulus fibers and vertebrae would reduce the stability of interbody spacers on a spinal segment, increasing cage stress on the interfaces under various combined loadings. This high stress might result in early failure of the endplate. Therefore, the stand-alone anterior two-part cage would not be indicated in the osteoporotic spine.

The results of FEA, as calculated by a mathematical method, should be interpreted as a trend only. This study has several limitations. First, inter-individual variation of bone geometry and material properties does not exactly reflect the behavior of all the human specimens tested. Major differences may occur, and validation of the results in an in vitro and in vivo study is recommended. Second, the bone-implant interface can be described as only an approximation to in vitro or in vivo conditions. It is unclear how the packed bone graft chips are connected to the host vertebrae. We have chosen to simulate the insertion of a solid spacer into the middle part of the disc space and do not think that this discrepancy from the in vitro model influenced our findings: the compressive load was mainly transferred through the peripheral part of the cages, not through the central part of the bone graft. Third, the interbody spacer was juxtaposed on an arbitrary shape/volume in the cancellous core of L4 and L5 vertebral body. We have assumed an ideal situation for graft incorporation without taking into account some factors: the effect of topology, stress at the graft/host bone interface, and local blood supply. Fourth, an extensive validation has not been done by comparing the prediction of several cage FEMs to a corresponding part of the experimental study, or by analyzing bone density to more precisely determined value of the osteoporotic level. We think that the influence of bone density on the compressive stiffness of cage FEA has been defined. The current FEA only predicts the relative movement of the segments under different loading conditions. For example, graft resorption, settling or partial implant failure may occur, resulting in a decrease of initial stability and the need for additional posterior instrumentation following cage insertion. Fifth, a fully bonded pedicle screw fixation was assumed on the posterior elements, which neglected the relaxation effect of the posterior implant on the coupling of load share after the solid arthrodesis. Finally, we have chosen to generate a L4–L5 model with material properties equivalent to those reported by Goel et al. [14,27,28]. Using experimental data from L2-3 and L3-4 human segments to validate a L4-5 model clearly is a limitation of the present study. However, with the consistent segmental lordosis and maximal contact of the cage-bone interface, it has been shown that both anatomical details and segmental flexibility are quite similar in human lumbar spine segments for L2-3, L3-4 or L4-5.

## Conclusion

In conclusion, the current study investigated the effects of geometric properties, loading conditions and cage-bone interface mechanics on the characteristics of several interbody cages. The biomechanical benefit of a stand-alone two-part cage is promising in spinal surgery to avoid surgical morbidities in damaged posterior muscles and facet joints caused by posterior instrumentation. This device addresses the stability required for interbody fusion, and supports the necessity of clinical trials using this alternative to the circumferential fixations. However, in the osteoporotic spine, supplementation with posterior fixation is recommended under various loading conditions.

## Competing interests

The authors declare that they have no competing interests.

## Authors' contributions

S–HC participated in the study design, in collecting the data, the statistically analyses and drafting of the manuscript. C–LT, C–YL and P–HH participated in the study design. W–PC advised and assisted drafting of the manuscript. All authors read and approved the final manuscript.

## Pre-publication history

The pre-publication history for this paper can be accessed here:


